# Sputum and Plasma Neutrophil Elastase in Stable Adult Patients With Cystic Fibrosis in Relation to Chronic Pseudomonas Aeruginosa Colonization

**DOI:** 10.7759/cureus.15948

**Published:** 2021-06-26

**Authors:** Atqah AbdulWahab, Mona Allangawi, Merlin Thomas, Ilham Bettahi, Siveen K Sivaraman, Jayakumar Jerobin, Prem Chandra, Manjunath Ramanjaneya, Abdul-Badi Abou-Samra

**Affiliations:** 1 Pediatric Pulmonology, Sidra Medicine, Doha, QAT; 2 Pulmonology, Hamad Medical Hospital, Doha, QAT; 3 Pulmonology, Hamad General Hospital, Doha, QAT; 4 Qatar Metabolic Institute, Interim Translational Research Institute, Academic Health System, Hamad General Hospital, Doha, QAT; 5 Interim Translational Research Institute, Academic Health System, Hamad General Hospital, Doha, QAT; 6 Medical Research Center, Hamad Medical Hospital, Doha, QAT; 7 Medicine, Hamad Medical Hospital, Doha, QAT

**Keywords:** cystic fibrosis, neutrophil elastase, inflammation, adults, pseudomonas aeruginosa

## Abstract

Background and purpose

Neutrophil elastase (NE) has been implicated in the pathogenesis of airway inflammation in cystic fibrosis (CF) patients and it impairs defenses against *Pseudomonas aeruginosa* (PA) infection or colonization. Sputum NE may act as a biomarker of neutrophilic inflammation in CF patients. This study aimed to determine sputum and plasma total NE levels in clinically stable adult CF patients and control subjects, and their correlation to PA colonization and lung functions.

Methods

This is a cross-sectional study. Total NE was measured on spontaneously expectorated sputum and plasma obtained from 21 CF patients, aged 18-40 years, during routine visits to the adult CF clinic. This was compared to plasma obtained from 22 matching healthy controls. The levels of NE were measured by the magnetic bead-based multiplex assay.

Results

Sputum and plasma NE levels had a significant positive correlation (Pearson r=0.533, P=0.013) with PA colonization. Sixteen CF patients (76.2%) were chronically colonized with PA. Both median sputum and plasma NE were found to be higher in CF patients with PA as compared with non-PA patients, even though this difference was statistically insignificant. Sputum and plasma NE levels did not correlate with the percentage predicted forced expiratory volume in one second (FEV1), the forced vital capacity (FVC), and FEV1/FVC and no association with PA.

Conclusion

The findings suggest that clinically stable adult CF patients colonized with PA may have higher NE levels in both plasma and sputum as compared to non-PA CF patients and probably total NE does not influence lung functions.

## Introduction

Cystic fibrosis (CF) is one of the most prevalent, life-shortening genetic diseases in the Caucasian population [1]. CF is caused by mutations in the cystic fibrosis transmembrane conductance regulator (CFTR) gene on chromosome 7, which encodes an epithelial anion channel that impacts multiple organ systems [2]. In CF airways, CFTR dysfunction or absence instigates the accumulation of abnormally thick, sticky mucus in the respiratory tract, which impairs bacterial mucociliary clearance and allows the colonization of the airways by microbial pathogens with *Pseudomonas aeruginosa* (PA) as the most relevant pathogen in the CF lung disease [3]. A systematic review reported that PA infection and pancreatic insufficiency were most commonly associated with lower baseline and more rapid declines in lung function [4].

Airway neutrophilia is a common feature of CF lung disease and is associated with disease progression, often regardless of the initiating cause. Neutrophils and their products are thought to be key mediators of the inflammatory changes in the airways of patients with CF [5].

Increased activity of neutrophil elastase (NE), a major product of activated neutrophils, has been identified as a key risk factor for the onset and progression of bronchiectasis and lung function decline in patients with and non-CF bronchiectasis [6,7]. NE is a 29-kD serine protease stored in azurophilic granules that may be released during degranulation, neutrophil extracellular trap formation, or cell death [8].

NE involvement may be direct (tissue damage) or indirect (proinflammatory or proapoptotic), or just be a marker of leukocyte activation [9]. NE has been implicated in the pathogenesis of mucus hypersecretion [10] and impaired defenses against PA infection [11]. It has been suggested that NE activity in sputum or bronchoalveolar lavage (BAL) fluid act as a promising biomarker of neutrophilic inflammation in CF lung disease [12]. 

The main objective of this study was to determine total NE levels in spontaneously expectorated sputum, and in plasma collected from clinically stable adults CF patients and healthy controls, and further investigate their association with PA colonization and correlation with lung function in adult CF patients.

This article was published as a preprint: AbdulWahab A, Allangawi M, Thomas M, et al.: Sputum and plasma adiponectin levels in clinically stable adult cystic fibrosis patients with CFTR I1234V mutation. Transl Med Commun. 2020, Vol. 5; p. 2. (https://transmedcomms.biomedcentral.com/articles/10.1186/s41231-020-00053-2)

## Materials and methods

This is a prospective cross-sectional study. The ethical committee of Hamad Medical Corporation (RC 16226/16) approved the study and informed consents were obtained from all subjects of the study. The total number of adult cystic fibrosis (CF ) patients in Qatar is 34 (18 males and 16 females), who were attending the adult CF clinics at Hamad Medical Corporation (HMC), Doha, Qatar. Twenty-one CF patients colonized with *Pseudomonas aeruginosa* (PA) in a stable condition and 22 control subjects were enrolled in the study. Both expectorated sputum and plasma were collected from CF patients and only plasma was collected from healthy control subjects who have no detected respiratory diseases. The diagnosis of CF was based on one or more clinical phenotypes consistent with CF, positive family history of CF in siblings and close relatives, confirmed by positive sweat chloride (> 60 mmol/l), and genetic analysis of the cystic fibrosis transmembrane regulator (CFTR) gene. Homozygous CFTR I1234V mutation is the most common CFTR mutation identified in Qatari CF patients belonging to a kindred of large Arab tribe and associated with pancreatic sufficiency among the majority of the patients [13,14].

The inclusion criteria included clinically stable adult (≥18 years old) CF patients, with no acute exacerbations or antibiotic use in the previous four weeks, who can expectorate sputum spontaneously. The exclusion criteria included current smokers and those who have quit smoking within the last two months of study, pregnant, lactating mothers, and patients who underwent lung transplants. Control subjects were chosen from bystanders reaching the clinic who are non-tobacco smokers and free of any infection for more than four weeks. The spontaneously expectorated sputum samples were collected for microbiological examination according to CF consensus guidelines and processed within two hours from collection [15]. As the expectorated sputum was considered the golden standard in the diagnosis of lower respiratory tract infections it was challenging while studying non-expectorating patients [16]. 

PA infection was defined as chronic when PA culture remains positive in the successive months in more than 50% of the preceding 12 months [17]. On the same day, the patients underwent Spirometric­­­­­­­­­­ evaluations, using Spiro bank, MIR, Italy, in accordance to the guidelines of the American Thoracic Society, which included the best recorded forced expiratory volume in one second (FEV1) as the maximum of three recorded measurements with < 15% variation, the forced vital capacity (FVC; in liters), forced expiratory volume in one second (FEV1; in liters), and FEV1/FVC (in %) as a percent of a predicted normal using standard equations [18]. 

Measurement of plasma and sputum neutrophil elastase

Plasma Processing

Whole blood was collected using commercially available ethylene-Diamine-Tetra-Acetic acid (EDTA) (BD Vacutainer K2E, BD Diagnostics, Oxford, UK) tubes. Centrifuged at 1000x*g* for 15 minutes within 30 minutes of collection. The resulting plasma supernatant was stored in different aliquots of 0.5 ml at -70°C until a sufficient number of samples were collected for various measurements.

Sputum Processing

All the sputum samples were collected in 50ml falcon tubes (Thermo Fisher Scientific, MA, USA). Approximately 1 g sputum sample was weighed and treated with freshly prepared 0.1% dithiothreitol (Sigma-Aldrich, Dorset, UK) at a ratio of 4:1 (v/w). The samples were incubated at 37°C for 10 minutes followed by vertex for five minutes. The resulting suspension was filtered through a 150 mesh wire net, centrifuged at 1500 rpm for 10 minutes. The resulting supernatant was transferred to an Eppendorf tube and stored at -70°C until required. Prior to NE measurement, all the supernatants were diluted with PBS 3 times for assessing NE levels in sputum samples. 

Total NE levels were measured using a magnetic bead-based Milli plex assay kit (Merck Millipore Corporation, Billerica, MA, USA; Cat# HSP3MAG-63K) as per the manufacturer’s recommended protocol. The detection range for NE was between 20 pg/mL and 20,000 pg/mL, the intra- and inter-assay variations were 6% and 7%, respectively. The plasma samples were diluted 100 times prior to the assay to get the NE levels within the detection range of the assay.

Statistical analysis

All statistical analyses performed using statistical packages SPSS version 27.0 (Armonk, NY: IBM Corp.) and Epi-info (Centers for Disease Control and Prevention, Atlanta, GA) software. Quantitative data were presented as mean ± standard deviation (SD) or median and ranges for skewed or non-normal data distribution. Categorical data values were expressed as frequencies and percentages. Pulmonary function parameters were expressed as mean ± SD. Statistical significances for quantitative data were computed using unpaired Student's t-test or Mann-Whitney U test for skewed data distribution. Pearson’s correlation was applied to determine the strength of the linear relationship between two or more quantitative variables (baseline concentrations of expectorated sputum and serum NE, in the CF patients and assess the relationship between FEV1 percentage, FVC percentage, and FEV1/FVC and association with PA). All P-values presented were two-sided, and P-values <0.05 were considered statistically significant.

## Results

Our study cohort consisted of 21 adult CF patients and 22 healthy controls. CF patient’s ages ranged between 18 years and 40 years with a mean age of 24.29±5.28 years. The main demographic and clinical characteristics are reported in Table 1. 

**Table 1:** Demographic and clinical data of CF and control subjects CF: cystic fibrosis; M: male; F: female; FEV1: forced expiratory volume in one second; FVC: forced vital capacity; NE: neutrophil elastase; CFTR: cystic fibrosis transmembrane conductance regulator; MRSA: methicillin-resistant staphylococcus aureus

	CF (N=21)	Control (N=22)	P-value
Gender (M/F), N	13/8	10/12	0.281
Age in years: mean (±SD)	24.29 ± 5.28	31.59 ± 10.37	0.003
CFTR genotype, N (%) homozygous CFTRI1234V mutation homozygous F508del/ F508del	20/21 (95.24%) 1/21 (4.76%)		
BMI (kg/m^2^): mean(±SD)	24.09±5.43	26.59 ± 3.93	0.092
Plasma NE: median (range) (ng/ml)	17.67 (0.79-340.22)	10.65 (0.91-29.57)	0.067
Sputum NE: median (range) (ng/ml)	3.78 (0.39-32.44)	-	-
*Pseudomonas aeruginosa* chronic colonized in lower respiratory secretion, N (%)	16/21 (76.2%)	-	-
MRSA, N (%)	1/21 (4.8%)	-	-
% Predicted FEV1: mean(±SD)	66.62 ± 20.04	-	-
% Predicted FVC: mean(±SD)	73.43 ± 16.21	-	-
% Predicted FEV1/ FVC: mean(±SD)	77.67 ± 11.749	-	-

**Figure 1:**
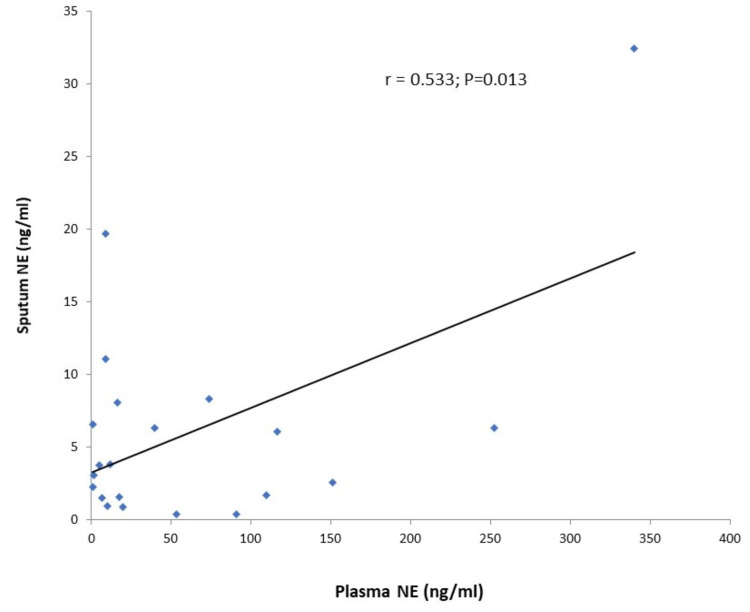
Correlation of sputum and plasma NE levels in CF subjects CF: cystic fibrosis; NE: neutrophil elastase

Thirteen CF patients had pancreatic sufficiency (62%), 16 CF patients (76.2%) were colonized with PA. The median (range) plasma NE in CF patients and control subjects were 17.67 (0.79-340.22) ng/ml and 10.65 (0.91-29.57) ng/ml (P=0.067), respectively. The median (range) sputum NE was 3.78 (0.39-32.44) ng/ml. A positive and significant correlation was observed between sputum and plasma NE (Pearson r=0.533, P=0.013) (Figure 1). 

Both median sputum and plasma NE levels were found to be higher in CF patients with chronic PA as compared with non-PA patients, even though this difference was not significant, The median (range) sputum NE in CF with PA 6.33 (0.39-32.44) ng/ml, versus non- PA 3.44(2.29-11.08) P=0.283 and the median (range) plasma NE in CF with PA was 18.68 (1.08-340.22), versus non-PA was 16.60 (0.80-252.25), P=0.869). Sputum and plasma NE were not significantly correlated (P>0.05) with percentage predicted FEV1, FVC, and FEV1/FVC, and there showed no association with PA colonization.

## Discussion

In the present study, the total plasma NE in expectorated sputum was analyzed to better characterize adult patients with CF in stable conditions who are infected with PA colonization. Sputum NE activity correlates with bronchiectasis in CF and the most informative sputum inflammatory biomarker to monitor CF lung disease both in stable state bronchiectasis, during exacerbations as well as in local or systemic antibiotic treatment [19]. 

In this study, the plasma NE levels in CF patients were not statistically different from those in healthy controls; however, there seemed to be a tendency for higher levels in CF patients (P=0.067).

NE is a major product released from neutrophils in inflamed airways, as a key risk factor for the onset and early progression of CF lung disease. However, the understanding of how NE and potentially other proteases contribute to the complex in vivo pathogenesis of CF lung disease remains limited [20]. Elevated NE activity in BAL fluid at three months of age was found to be associated with persistent bronchiectasis by the Australian Respiratory Early Surveillance Team for cystic fibrosis [21].

It is known that chronic PA bronchial infection causes an inflammatory trigger in both CF and bronchiectasis [22,23]. In our study, we found that median CF sputum and CF plasma NE were not significantly different in PA and non-PA groups, however, a tendency for higher values was observed in PA positive group as compared to PA negative group. Sagel et al. demonstrated higher neutrophil count, IL-8, and NE levels in BAL samples of patients with CF harbor PA as compared to those with negative for PA and positive for other pathogens [24]. Another study, demonstrated in clinically stable conditions, that CF patients with chronic PA infection were known to have elevated levels of inflammatory biomarkers compared to the non-CF population [25].

FEV1 is the gold standard for monitoring disease progression and a surrogate marker to determine the degree of severity of airway CF lung disease. The present study does not correlate total NE with the percentage predicted FEV1, FVC, and FEV1/FVC both in sputum and plasma samples. Indeed, free NE in sputum has long been known to correlate with FEV1 in children with CF [26]. Another study reported that FEV1 inversely correlated with total cell count and free NE in BAL fluid obtained from patients with CF [27]. 

Our study has a few limitations. First, this study was designed to be cross-sectional that does not provide information on the long-term relationship between sputum and plasma NE and lung function changes or chronic PA colonization in adult CF patients. Second, this study pays attention to limited measurement of total NE in both expectorated sputum and plasma and not active NE. Third, the limitation of this study is that it included expectorated sputum and excluded non expectorated in CF and healthy subjects. The fourth limitation is the small sample size owing to the small number of CF patients in Qatar.

## Conclusions

The present study suggests that clinically stable adult CF patients colonized with PA may have higher NE levels in either plasma or sputum as compared to non-PA CF patients and probably total NE may not have an influence on lung functions. Further, prospective prolonged follow-up may be required to detect the association of lung function in CF patients colonized with PA and the impact of both total and active NE in both sputum and plasma

## References

[1] O’Sullivan BP, Freedman SD (2009). Cystic fibrosis. Lancet.

[2] Rowe SM, Miller S, Sorscher EJ (2005). Cystic fibrosis. N Engl J Med.

[3] Bhagirath AY, Li Y, Somayajula D, Dadashi M, Badr S, Duan K (2016). Cystic fibrosis lung environment and Pseudomonas aeruginosa infection. BMC Pulm Med.

[4] Harun SN, Wainwright C, Klein K, Hennig S (2016). A systematic review of studies examining the rate of lung function decline in patients with cystic fibrosis. Paediatr Respir Rev.

[5] Jasper AE, McIver WJ, Sapey E, Walton GM (2019). Understanding the role of neutrophils in chronic inflammatory airway disease. F1000Res.

[6] Chalmers JD, Moffitt KL, Suarez-Cuartin G (2017). Neutrophil elastase activity is associated with exacerbations and lung function decline in bronchiectasis. Am J Respir Crit Care Med.

[7] Mayer-Hamblett N, Aitken ML, Accurso FJ (2007). Association between pulmonary function and sputum biomarkers in cystic fibrosis. Am J Respir Crit Care Med.

[8] Gifford AM, Chalmers JD (2014). The role of neutrophils in cystic fibrosis. Curr Opin Hematol.

[9] Polverino E, Rosales-Mayor E, Dale GE, Dembowsky K, Torres A (2017). The role of neutrophil elastase inhibitors in lung diseases. Chest.

[10] Lee WL, Downey GP (2001). Leukocyte elastase: physiological functions and role in acute lung injury. Am J Respir Crit Care Med.

[11] Weldon S, McNally P, McElvaney NG (2009). Decreased levels of secretory leucoprotease inhibitor in the Pseudomonas-infected cystic fibrosis lung are due to neutrophil elastase degradation. J Immunol.

[12] Dittrich AS, Kühbandner I, Gehrig S (2018). Elastase activity on sputum neutrophils correlates with severity of lung disease in cystic fibrosis. Eur Respir J.

[13] Abdul Wahab A, Al Thani G, Dawod ST, Kambouris M, Al Hamed M (2001). Heterogeneity of the cystic fibrosis phenotype in a large kindred family in Qatar with cystic fibrosis mutation (I1234V). J Trop Pediatr.

[14] Abdel Rahman H, Abdul Wahab A, Abdel Rahman MO, Mostafa OA (2006). Faecal elastase-1 concentration in cystic fibrosis patients with CFTR I1234V mutation. Acta Paediatr.

[15] (2010). Laboratory standards for processing microbiological samples from people with cystic fibrosis. First edition. September.

[16] Eyns H, Piérard D, De Wachter E, Eeckhout L, Vaes P, Malfroot A (2018). Respiratory bacterial culture sampling in expectorating and non-expectorating patients with cystic fibrosis. Front Pediatr.

[17] Lee TW, Brownlee KG, Conway SP, Denton M, Littlewood JM (2003). Evaluation of a new definition for chronic Pseudomonas aeruginosa infection in cystic fibrosis patients. J Cyst Fibros.

[18] (1995). Standardization of spirometry, 1994 update. American Thoracic Society. Am J Respir Crit Care Med.

[19] DeBoer EM, Swiercz W, Heltshe SL (2014). Automated CT scan scores of bronchiectasis and air trapping in cystic fibrosis. Chest.

[20] Wagner CJ, Schultz C, Mall MA (2016). Neutrophil elastase and matrix metalloproteinase 12 in cystic fibrosis lung disease. Mol Cell Pediatr.

[21] Sly PD, Gangell CL, Chen L (2013). Risk factors for bronchiectasis in children with cystic fibrosis. N Engl J Med.

[22] Schaaf B, Wieghorst A, Aries SP, Dalhoff K, Braun J (2000). Neutrophil inflammation and activation in bronchiectasis: comparison with pneumonia and idiopathic pulmonary fibrosis. Respiration.

[23] Tsang KW, Chan K, Ho P, Zheng L, Ooi GC, Ho JC, Lam W (2000). Sputum elastase in steady-state bronchiectasis. Chest.

[24] Sagel SD, Gibson RL, Emerson J (2009). Impact of Pseudomonas and Staphylococcus infection on inflammation and clinical status in young children with cystic fibrosis. J Pediatr.

[25] Jones AM, Martin L, Bright-Thomas RJ (2003). Inflammatory markers in cystic fibrosis patients with transmissible Pseudomonas aeruginosa. Eur Respir J.

[26] Sagel SD, Sontag MK, Wagener JS, Kapsner RK, Osberg I, Accurso FJ (2002). Induced sputum inflammatory measures correlate with lung function in children with cystic fibrosis. J Pediatr.

[27] Pillarisetti N, Williamson E, Linnane B (2011). Infection, inflammation, and lung function decline in infants with cystic fibrosis. Am J Respir Crit Care Med.

